# Low Expression of ADCY4 Predicts Worse Survival of Lung Squamous Cell Carcinoma Based on Integrated Analysis and Immunohistochemical Verification

**DOI:** 10.3389/fonc.2021.637733

**Published:** 2021-06-10

**Authors:** Zhicong Liu, Lixin Ru, Zhenchao Ma

**Affiliations:** ^1^ Department of Respiratory Medicine, Affiliated Huzhou Hospital, Zhejiang University School of Medicine, Huzhou Central Hospital, Affiliated Central Hospital Huzhou University, Huzhou, China; ^2^ Department of Radiation Oncology, Affiliated Huzhou Hospital, Zhejiang University School of Medicine, Huzhou Central Hospital, Affiliated Central Hospital Huzhou University, Huzhou, China; ^3^ Department of Radiation Oncology, The Second Affiliated Hospital of Soochow University, Suzhou, China

**Keywords:** lung squamous cell carcinoma, differential gene expression analysis, weighted gene co-expression network analysis, protein-protein interaction network, immunohistochemistry, survival analysis

## Abstract

**Purpose:**

The molecular mechanism underlying the carcinogenesis and development of lung squamous cell carcinoma (LUSC) has not been sufficiently elucidated. This analysis was performed to find pivotal genes and explore their prognostic roles in LUSC.

**Methods:**

A microarray dataset from GEO (GSE19188) and a TCGA-LUSC dataset were used to identify differentially co-expressed genes through Weighted Gene Co-expression Network Analysis (WGCNA) and differential gene expression analysis. We conducted functional enrichment analyses of differentially co-expressed genes and established a protein-protein interaction (PPI) network. Then, we identified the top 10 hub genes using the Maximal Clique Centrality (MCC) algorithm. We performed overall survival (OS) analysis of these hub genes among LUSC cases. GSEA analyses of survival-related hub genes were conducted. Ultimately, the GEO and The Human Protein Atlas (THPA) databases and immunohistochemistry (IHC) results from the real world were used to verify our findings.

**Results:**

A list of 576 differentially co-expressed genes were selected. Functional enrichment analysis indicated that regulation of vasculature development, cell−cell junctions, actin binding and PPAR signaling pathways were mainly enriched. The top 10 hub genes were selected according to the ranking of MCC scores, and 5 genes were closely correlated with OS of LUSC. Additionally, GSEA analysis showed that spliceosome and cell adhesion molecules were associated with the expression of GNG11 and ADCY4, respectively. The GSE30219 and THPA databases and IHC results from the real world indicated that although GNG11 was not detected, ADCY4 was obviously downregulated in LUSC tissues at the mRNA and protein levels.

**Conclusions:**

This analysis showed that survival-related hub genes are highly correlated to the tumorigenesis and development of LUSC. Additionally, ADCY4 is a candidate therapeutic and prognostic biomarker of LUSC.

## Introduction

Lung carcinoma is a common cancer, with nearly 228820 cancer patients and 135720 deaths in 2020, which places an enormous burden on patients and their families (1). Non-small cell lung cancer (NSCLC) accounts for approximately 85% of all cases of lung carcinoma, and the most common pathological pattern of NSCLC is lung adenocarcinoma (LUAD), followed by lung squamous cell carcinoma (LUSC) (2). In recent years, many studies have suggested that some molecular abnormalities are associated with cell proliferation, invasion and poor survival of LUSC (3). Compared to the strategies for LUSC, strategies for the early diagnosis and therapy of LUSC remain highly limited (4). Therefore, it is essential to find important biomarkers for the occurrence and adverse progression of LUSC, which will greatly accelerate the development of useful therapeutic strategies.

With the speedy development of genomic technology, researchers have analyzed gene expression profiles using bioinformatics approaches to explore the underlying molecular mechanisms of tumors and detect cancer-specific biomarkers (5). Weighed Gene Co-expression Network Analysis (WGCNA) is an important algorithm to understand gene co-expression networks and gene functions (6). Using WGCNA, we can detect the modules of closely correlated genes related to the traits of samples, which will provide insights to predict probable functions of co-expression genes (7). In addition, differential gene expression analysis is usually applied for the analysis of transcriptomics datasets, which is beneficial to explore underlying biological and molecular mechanisms of cancers and detect quantitative differences between the gene expression levels of intervention and control cohorts (8).

To achieve a higher capability to discriminate closely related genes, we used the two approaches mentioned above in our analysis. First, gene expression profiles of LUSC were obtained from Gene Expression Omnibus (GEO) and The Cancer Genome Atlas database (TCGA). Second, we used WGCNA and differential gene expression analysis to identify common differentially co-expressed genes. Next, functional enrichment analysis, protein-protein interaction (PPI) analysis and overall survival (OS) analysis were carried out to detect candidate indicators related to the carcinogenesis and adverse invasion of LUSC. Then, gene set enrichment analysis (GSEA) of survival-related hub genes was conducted using the TCGA-LUSC dataset. Finally, we validated the mRNA and protein expression levels of OS-related hub genes through GEO, The Human Protein Atlas (THPA) and immunohistochemistry (IHC) results from the real world.

## Materials and Methods

**Figure 1:**
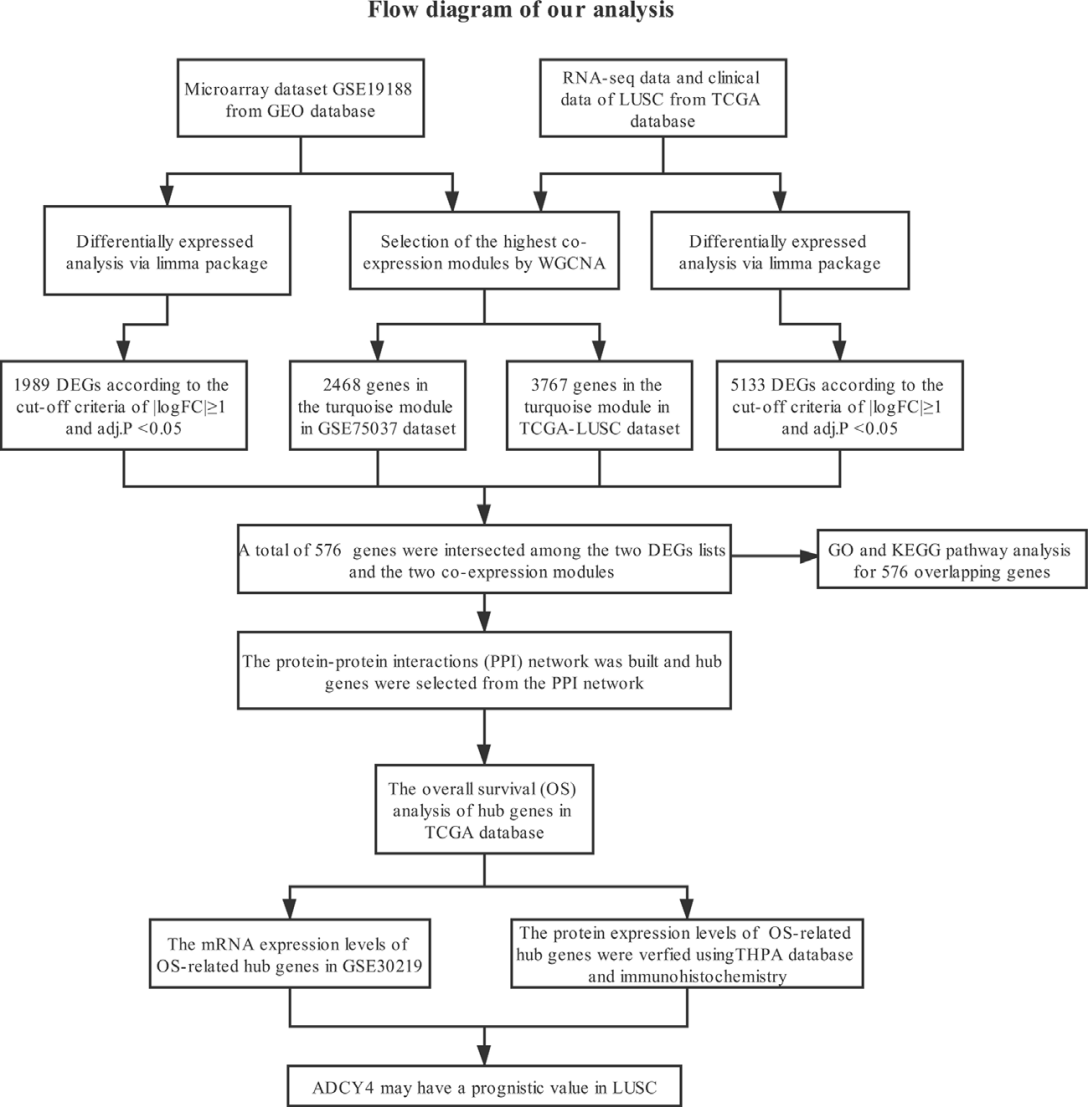
Study design and workflow of our study.

**Figure 1** shows the specific steps including dataset download, hub gene identification and external verification of LUSC. Each procedure will be described in the following sub-sections.

### Dataset Download

Gene expression profiles of LUSC were acquired from the GEO and TCGA databases. First, GSE19188 was obtained from GEO for further analysis, containing 27 LUSC and 65 normal lung tissues. GSE19188 is based on [HG-U133_Plus_2] Affymetrix Human Genome U133 Plus 2.0 Array. Using the annotation file provided by the manufacturer, probes were switched to corresponding gene symbols, probes without gene symbols were deleted, and several probes of the same gene were averaged. In total, we obtained 21655 genes for subsequent analysis. Second, we downloaded the gene expression dataset and clinical information of LUSC from TCGA database. We acquired 551 samples from TCGA (**Table S1**), including 502 LUSC and 49 normal lung tissues, as well as RNA-Seq fragments per kilobase per million (FPKM) data of 19645 genes. Furthermore, we transformed FPKM format to transcript per million (TPM) format for our subsequent analysis. Based on the Illumina HiSeq 2000 platform, all data were generated and annotated to a reference transcript set: Human hg38 gene standard track. The edgeR package tutorial suggests that genes with low-read counts commonly play insignificant roles in subsequent analysis (9). Thus, we removed genes with TPM<1 from our analysis, and we acquired 15153 genes for the following analysis.

### Selection of Important Co-Expression Modules Using WGCNA

**Figure 2:**
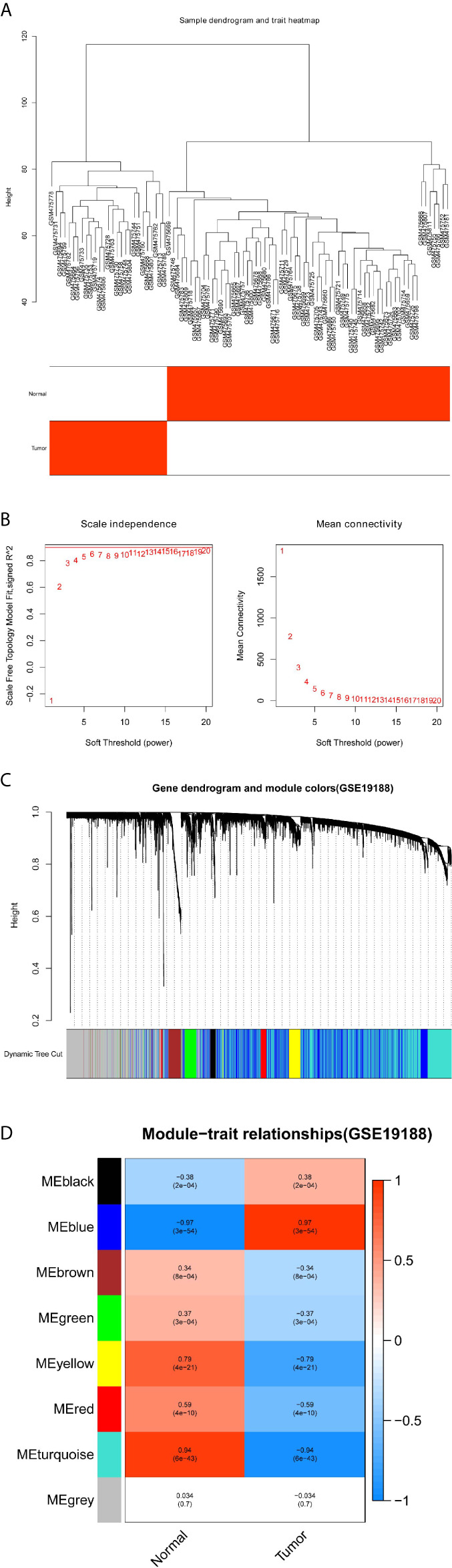
Identification of modules correlated with the clinical traits in GSE19188. **(A)** Sample dendrogram and trait heatmap. **(B)** Scale independence and Mean connectivity. **(C)** The Cluster dendrogram of co-expression network modules is ordered by a hierarchical clustering of genes based on the 1-TOM matrix. Different colors represent different modules. **(D)** Module-trait relationships. Each row represents a color module and every column represents a clinical trait (normal and tumor). Each cell contains the corresponding correlation and P-value.

**Figure 3:**
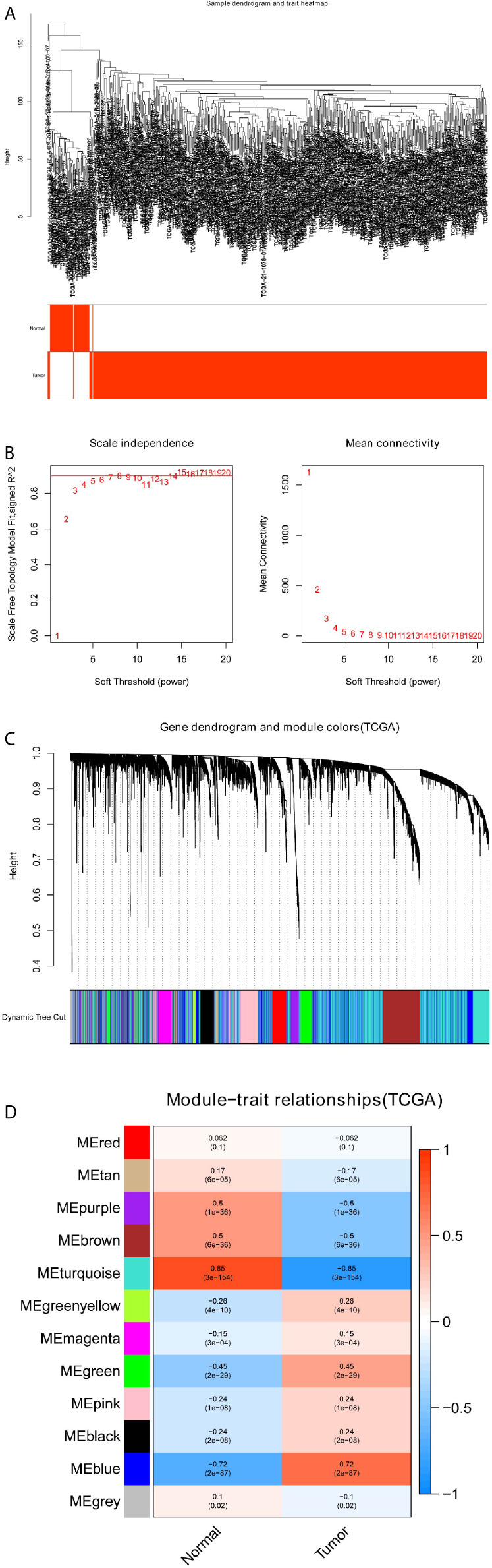
Identification of modules correlated with the clinical traits in TCGA-LUSC dataset. **(A)** Sample dendrogram and trait heatmap. **(B)** Scale independence and Mean connectivity. **(C)** The Cluster dendrogram of co-expression network modules is ordered by a hierarchical clustering of genes based on the 1-TOM matrix. Different colors represent different modules. **(D)** Module-trait relationships. Each row represents a color module and every column represents a clinical trait (normal and tumor). Each cell contains the corresponding correlation and P-value.

The gene co-expression networks of GSE19188 and TCGA-LUSC dataset were built through the WGCNA package (6). WGCNA can find closely related genes and aggregate these genes into the same co-expression module correlated with clinical traits. To establish a scale-free network, we used soft powers β=11 (**Figures 2A, B**) and 5 (**Figures 3A, B**) for the GSE19188 and TCGA-LUSC datasets. Next, we created an adjacency matrix with the following formula: aij = |Sij|^β^ (aij: adjacency matrix between gene i and gene j, Sij: similarity matrix that is done by Pearson correlation of all gene pairs, β: soft power value), and we converted this matrix to a topological overlap matrix (TOM) and its corresponding dissimilarity (1-TOM). The hierarchical clustering dendrogram of the 1-TOM matrix was established to aggregate genes with similar expressions into one co-expression module. Afterward, we explored the module-trait relations between modules and external traits to find functional modules in this co-expression network. The module with the highest correlation coefficient is believed to be the candidate module that is closely correlated with clinical traits, and we used this module for our subsequent analysis.

### Identification of Differentially Co-Expressed Genes

The limma package is usually adopted to conduct differential gene expression analysis of microarray and RNA-Seq datasets (10). The limma package was used for the differential expression analysis of the GSE19188 and TCGA-LUSC datasets to obtain differentially expressed genes (DEGs) between LUSC and normal lung tissues. To decrease the false discovery rate (FDR), we adjusted the P-value through the Benjamini–Hochberg method. The selection criteria for DEGs were |logFC|≥1 and adj.P <0.05. Subsequently, to improve the capability to discriminate closely related genes, the intersections between the two lists of DEGs and the two lists of co-expression genes from the two co-expression networks were taken as common genes, and these common genes were applied to find potential prognostic indicators of LUSC.

### Functional Enrichment Analysis of Differentially Co-Expressed Genes

Functional enrichment analysis includes two components, namely, gene ontology (GO) as well as Kyoto Encyclopedia of Genes and Genomes (KEGG) pathway analyses. To analyze their biological functions, we performed GO and KEGG pathway analyses of differentially co-expressed genes using the clusterProfiler (11) and GOplot packages. GO is a notable bioinformatics tool applied to annotate genes and explore their biological processes (12). GO enrichment analysis includes biological processes (BP), cellular component (CC) as well as molecular function (MF). KEGG is helpful to understand high-level functions and biological systems from large-scale molecular datasets (13). P<0.05 is regarded as significantly different.

### PPI Network Construction and Hub Gene Selection

The PPI network of differentially co-expressed genes was established through the Search Tool for the Retrieval of Interacting Genes (STRING) database (14). Cytoscape was applied to establish a visual network of molecular interactions with the combined score>0.6 (15). The plugin Molecular Complex Detection (MCODE) was applied to detect highly correlated modules from PPI networks (16). The most significant gene module from the PPI network was visualized and shown using the MCODE plug-in. The criteria for filtering were: MCODE score >5, node score cut-off =0.2, degree cut-off =2, k-score =2 as well as Max depth =100. Furthermore, the Maximal Clique Centrality (MCC) algorithm is one of the most useful approaches to select hub nodes from PPI networks (17). The MCC values of all genes in the PPI network were calculated through the CytoHubba plugin. We considered the top 10 genes with the highest MCC scores as hub genes. Also, we visualized these hub genes *via* the CytoHubba plugin.

### Overall Survival of Hub Genes

To explore the prognostic roles of the top ten hub genes, we conducted Kaplan–Meier univariate survival analysis through the survival package based on the TCGA-LUSC dataset. LUSC cases without completed follow-up information (n=6) were excluded from the survival analysis, and then other patients from the TCGA-LUSC dataset were classified into two cohorts according to the median expression levels of hub genes. Log-rank p<0.05 is considered statistically significant.

### GSEA Analysis of Survival-Related Hub Genes

As an important computing method, GSEA recognizes if a previously defined gene set is statistically significant and concordantly different between two biological states (18). LUSC samples were stratified into two cohorts according to the median expression values of survival-related hub genes. Next, we analyzed the effects of their expression on some gene sets to obtain related KEGG pathways through the molecular signatures database (MSigDB) (c2.cp.kegg.all.v7.1.symbols.gmt) (19). The permutation of every analysis was repeated 1000 times. |Normalized enrichment score (NES)|> 1, nominal (NOM) p-value<0.05 and FDR q-value <0.25 were regarded as significantly different.

### External Validation of the GEO and THPA Databases

To increase the reliability of our analysis, the GEO and THPA databases were used to verify the expression levels of survival-related hub genes between LUSC and normal lung tissues. We explored the mRNA expression levels of these hub genes between LUSC and non-malignant adjacent tissues using GSE30219 from GEO. Furthermore, we explored the protein expression patterns of these hub genes between LUSC and non-malignant adjacent tissues using IHC from the THPA database (20).

### Immunohistochemistry Based on the Real World

To improve the reliability of our findings, LUSC and normal lung tissue samples were acquired from the Huzhou Central Hospital (Zhejiang, China). We performed IHC staining on tissue slices from paraffin-embedded tissues, which was approved by Medical Ethics Committee of Huzhou Central Hospital. We mounted tissue slices on glass microscope slides, deparaffinized with dimethyl benzene, and rehydrated using graded ethanol. Then, we carried out antigen retrieval at a high temperature in a water bath. Subsequently, we cooled, rinsed, and quenched the endogenous peroxidases of slides with 3% hydrogen peroxide. Afterward, slides were incubated with 5% BSA for 45 min at room temperature, and the slides were incubated overnight with anti-ADCY4 and anti-GNG11 antibodies (dilutions: 1:150 and 1:350, respectively; Sigma, USA). We washed and incubated these slides with secondary antibody for one hour. The protein expression of ADCY4 and GNG11 was evaluated semiquantitatively according to total scores of the area of positive-stained cells and staining intensity. The area of positive-stained cells was scored as 0 = 0~10%, 1 = 10% to 25%, 2 = 25% to 50%, 3 = 50% to 75% and 4 = 75% to 100%, while the staining intensity was scored as: 0=negative, 1=weakly, 2=moderately, 3=strongly. Independent scores were estimated by two pathologists, and mean scores were considered the final immunostaining scores. When final immunostaining scores were larger than 2, the tissue samples were considered highly expressed; otherwise, the samples had low expression (21).

## Results

### Identification of Important Co-Expression Modules Using WGCNA

To detect the functional modules in LUSC, we established two gene co-expression networks through the WGCNA package based on the GSE19188 and TCGA-LUSC datasets, respectively. We found 8 modules in the GSE19188 dataset (**Figure 2C**) and 12 modules in the TCGA-LUSC dataset (**Figure 3C**). Afterward, the two heatmaps explored the relationship between these modules and two clinical traits (normal lung and LUSC tissues) in the GSE19188 (**Figure 2D**) and TCGA-LUSC datasets (**Figure 3D**), suggesting that the turquoise module in GSE19188 and the turquoise module in the TCGA-LUSC dataset were highly correlated with normal lung tissues (turquoise module in GSE19188: r=0.94, P=6e-43; turquoise module in TCGA-LUSC dataset: r=0.85, P=3e-154).

### Selection of Differentially Co-Expressed Genes

**Figure 4:**
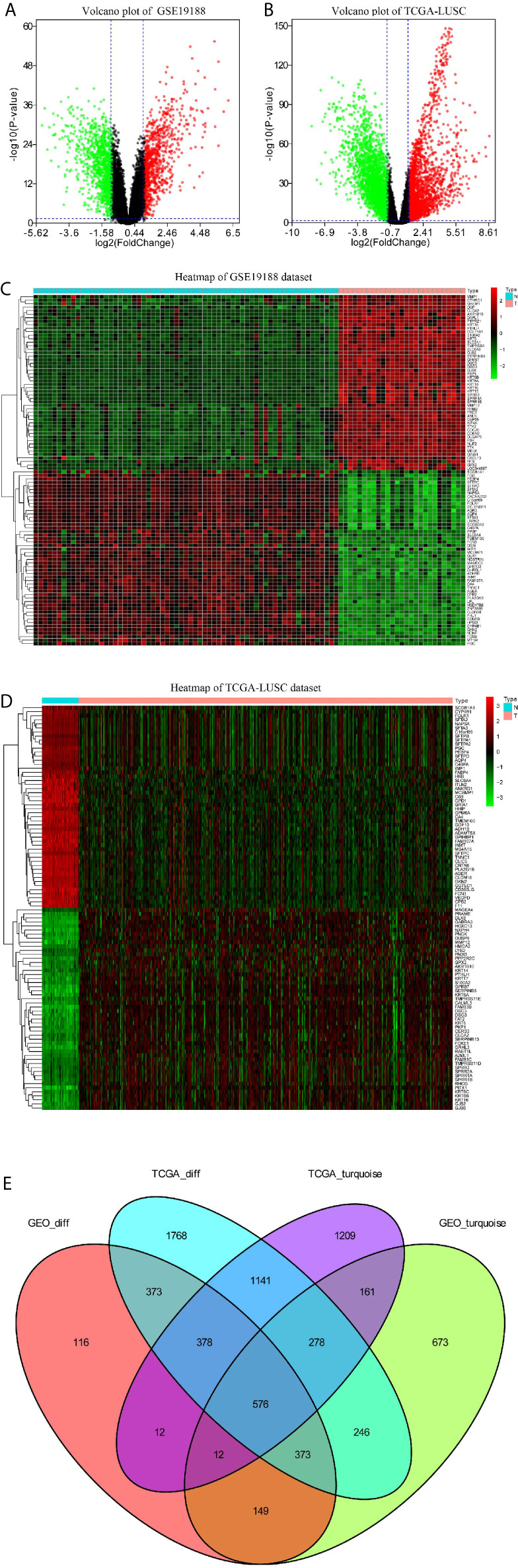
Identification of differentially expressed genes (DEGs) among GSE19188 TCGA-LUSC dataset with the cut-off criteria of |logFC|>1 and adj.P <0.05. **(A)** Heatmap of top 50 upregulated and 50 downregulated DEGs of GSE19188. **(B)** Heatmap of top 50 upregulated and 50 downregulated DEGs of TCGA-LUSC dataset. **(C)** Volcano plot of DEGs in GSE19188. **(D)** Volcano plot of DEGs in the TCGA-LUSC dataset. **(E)** The Venn diagram of genes among the two DEG lists and the two lists of co-expression genes. In total, 576 overlapping differential co-expression genes are found.

The volcano plots revealed that 1989 DEGs in GSE19188 (**Figure 4A**) and 5133 DEGs in TCGA-LUSC dataset (**Figure 4B**) were obviously dysregulated between LUSC and non-malignant adjacent tissues. The heatmaps illustrated the expression patterns of 50 upregulated and 50 downregulated genes in the GSE19188 (**Figure 4C**) and TCGA-LUSC datasets (**Figure 4D**). **Figure 4E** clearly shows the intersection of two lists of DEGs (**Tables S2** and **S3**) and two lists of co-expression genes (**Tables S4** and **S5**), which included a total of 576 genes (**Table S6**) that were applied for our next analysis.

### Functional Enrichment Analysis

**Figure 5:**
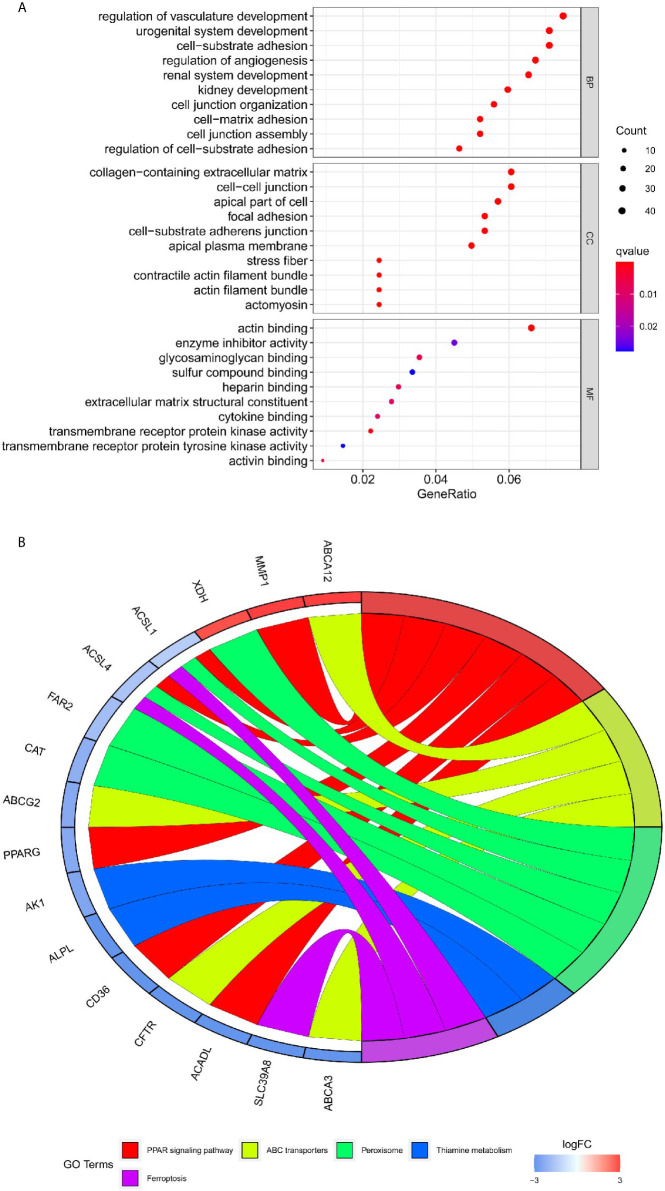
Functional enrichment analysis of differential co-expression genes using the clusterProfiler package. **(A)** Gene ontology (GO) enrichment analysis of differential co-expression genes. The color represents the adjusted P-value, and the size of the spots represents the gene number. **(B)** Kyoto encyclopedia of genes and genomes pathway (KEGG) of differential co-expression genes.

To acquire further insights into potential biological functions, GO and KEGG pathway analyses of these differentially co-expressed genes were conducted. We observed that BP analysis of the 576 genes was primarily enriched for the regulation of vasculature development and cell-substrate adhesion. The CC analysis suggested that collagen−containing extracellular matrix and cell−cell junction were associated with the 576 genes. According to the results of MF analysis, actin binding and enzyme inhibitor activity were mainly enriched (**Figure 5A**). Additionally, KEGG pathway analysis showed that PPAR signaling pathway and ABC transporters were significantly enriched (**Figure 5B**).

### PPI Network Construction and Hub Gene Selection

**Figure 6:**
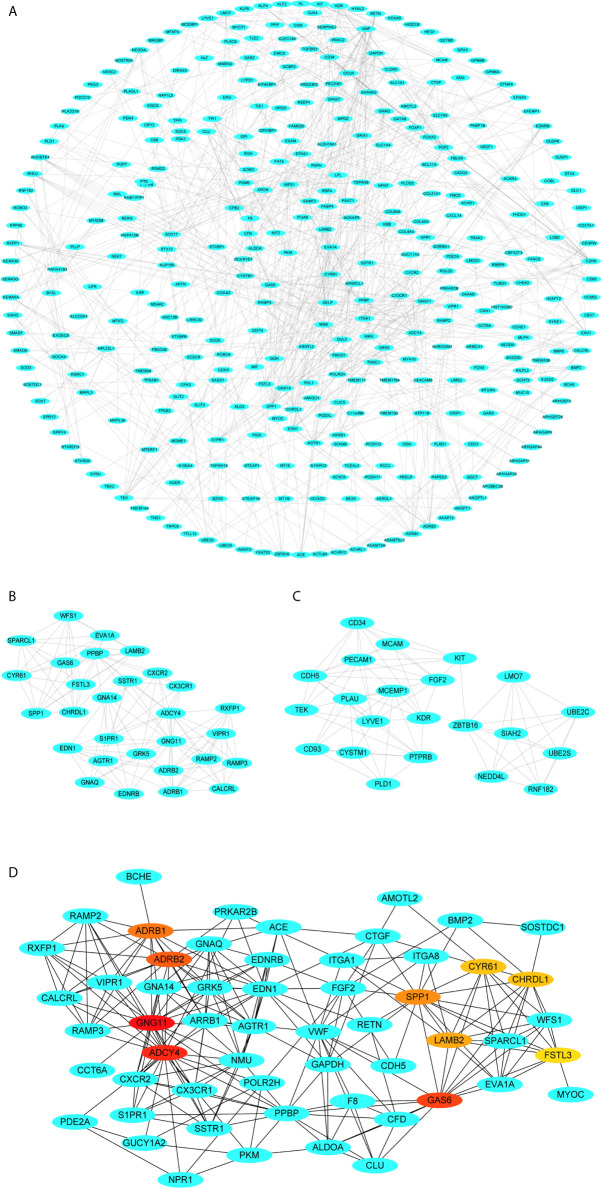
Visualization of the protein-protein interaction (PPI) network, the most significant modules and hub genes. **(A)** PPI network of differential co-expression genes. **(B)** The most significant module from PPI network. **(C)** The second most significant module from PPI network. **(D)** Selection of hub genes from PPI network through maximal clique centrality (MCC) algorithm. The turquoise nodes represent the genes. Edges suggest the protein-protein relations. The red nodes represent genes with high MCC values, whereas the yellow nodes represent genes with low MCC values.

The PPI network of these genes with 357 nodes and 744 edges is clearly shown (**Figure 6A** **)**. The most significant module was detected using the MCODE plugin, containing 29 nodes and 124 edges (**Figure 6B**). Also, the second most significant module was detected, including 22 nodes and 71 edges (**Figure 6C**). Subsequently, genes with top ten highest MCC scores were designated as hub genes (GNG11, ADCY4, GAS6, ADRB2, ADRB1, SPP1, LAMB2, CYR61, CHRDL1 and FSTL3). The top ten hub genes from this PPI network are vividly displayed, and the color shade represents the magnitude of the MCC values of hub genes (**Figure 6D**).

### Prognostic Roles of Hub Genes

**Figure 7:**
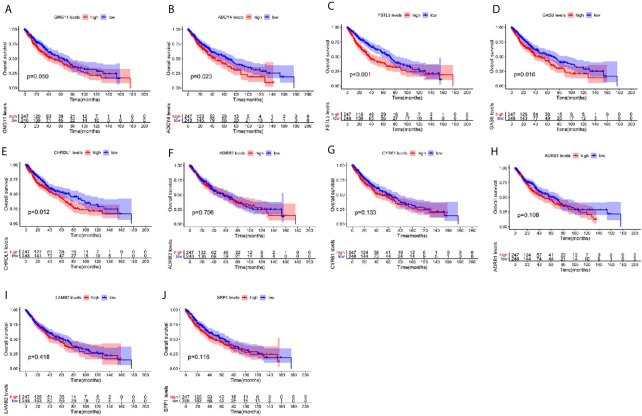
Overall survival (OS) analysis of the top 10 hub genes among patients from TCGA-LUSC dataset. Survival analysis for **(A)** GNG11, **(B)** ADCY4, **(C)** FSTL3, **(D)** GAS6, **(E)** CHRDL1, **(F)** ARDB2, **(G)** CYR61, **(H)** ARDB1, **(I)** LAMB2, and **(J)** SPP1 in LUSC. The LUSC patients are divided into high expression cohort (red) and low expression cohort (blue) according to the median expression of hub genes. Log-rank P ≤ 0.05 is believed as statistical difference.

To explore the prognostic roles of the top 10 hub genes in LUSC, we conducted overall survival analysis of the top 10 hub genes using the clinical information from the TCGA-LUSC dataset (**Figures 7A–J**). Five hub genes were found to be highly correlated with the survival of patients with LUSC, namely, the higher expression of GNG11, ADCY4, FSTL3, GAS6, and CHRDL1 was significantly correlated with worse survival of LUSC (**Figures 7A–E**).

### GSEA Analysis of Survival-Related Hub Genes

**Figure 8:**
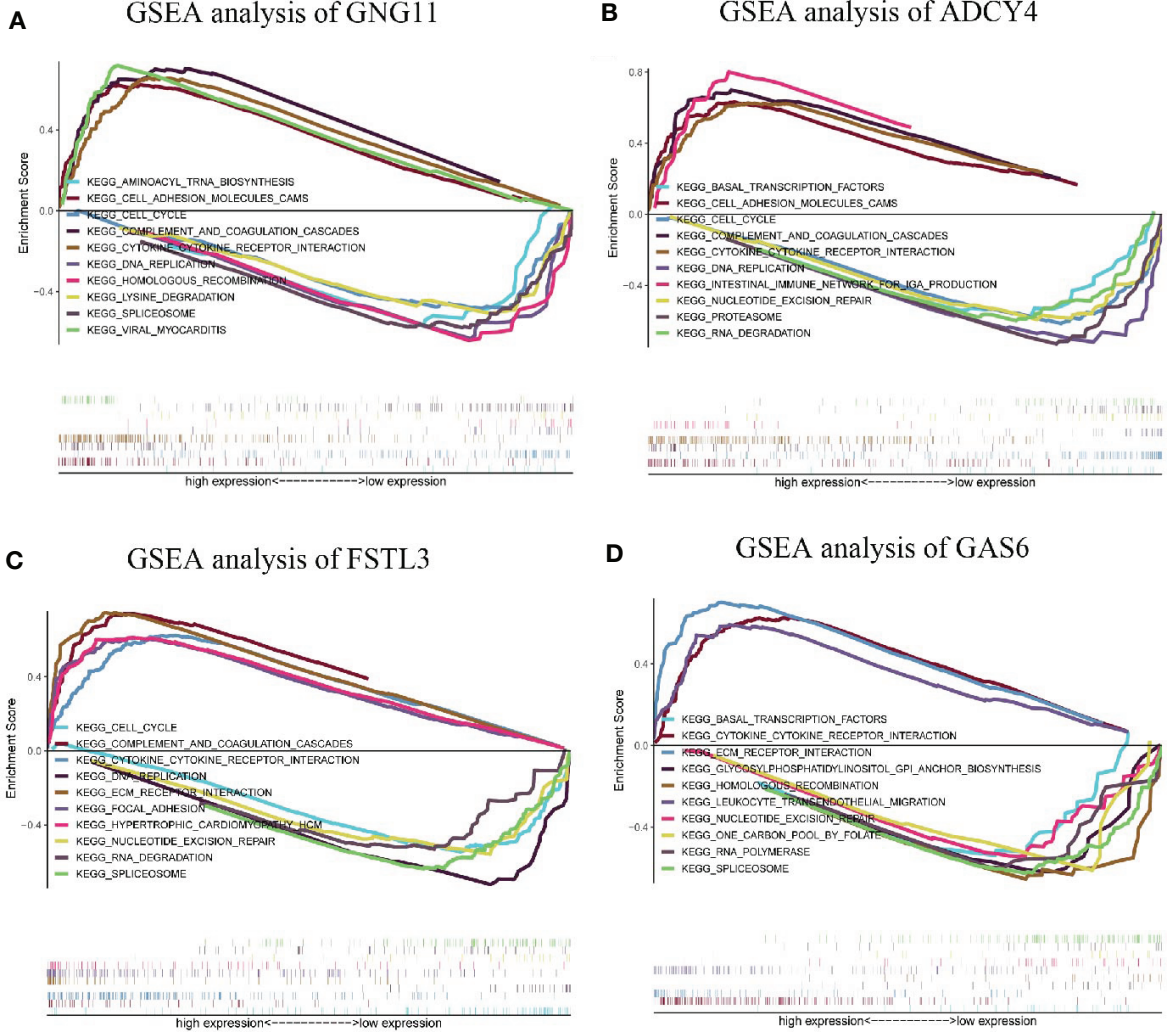
Enrichment plots by Gene Set Enrichment Analysis (GSEA). Relative pathways associated with the expression of **(A)** GNG11, **(B)** ADCY4, **(C)** FSTL3, and **(D)** GAS6 are displayed.

GSEA analysis demonstrated that spliceosome and viral myocarditis were correlated with GNG11 (**Figure 8A**). GSEA analysis showed that cell adhesion molecules (cams) and the cell cycle were associated with ADCY4 (**Figure 8B**). Furthermore, GSEA analysis revealed that spliceosome and ECM receptor interaction were associated with FSTL3 expression (**Figure 8C**). GSEA analysis suggested that cytokine-cytokine receptor interactions and one carbon pool modulated by folate were correlated with GAS6 expression (**Figure 8D**). However, the GSEA analysis of CHRDL1 revealed that no KEGG pathway met our selection criteria. In detail, the results of the GSEA analysis are shown in **Table 1** and **Table S7**.

**Table 1:** Relative pathways associated with the expression of GNG11 and ADCY4 using GSEA.

Gene	Name	ES	NES	NOM p-value	FDR q-value
GNG11	KEGG_LYSINE_DEGRADATION	-0.54	-1.92	<0.0001	0.101
	KEGG_SPLICEOSOME	-0.59	-1.89	0.002	0.077
	KEGG_HOMOLOGOUS_RECOMBINATION	-0.67	-1.84	0.008	0.086
	KEGG_CELL_CYCLE	-0.49	-1.76	0.004	0.142
	KEGG_AMINOACYL_TRNA_BIOSYNTHESIS	-0.61	-1.69	0.014	0.202
	KEGG_DNA_REPLICATION	-0.66	-1.67	0.031	0.204
	KEGG_CYTOKINE_CYTOKINE_RECEPTOR_INTERACTION	0.66	2.21	<0.0001	0.002
	KEGG_VIRAL_MYOCARDITIS	0.72	2.17	<0.0001	0.001
	KEGG_CELL_ADHESION_MOLECULES_CAMS	0.62	2.08	<0.0001	0.003
	KEGG_COMPLEMENT_AND_COAGULATION_CASCADES	0.70	2.05	<0.0001	0.004
ADCY4	KEGG_CYTOKINE_CYTOKINE_RECEPTOR_INTERACTION	0.63	2.18	<0.0001	<0.0001
	KEGG_CELL_ADHESION_MOLECULES_CAMS	0.63	2.16	<0.0001	<0.0001
	KEGG_COMPLEMENT_AND_COAGULATION_CASCADES	0.70	1.99	0.002	0.009
	KEGG_INTESTINAL_IMMUNE_NETWORK_FOR_IGA_PRODUCTION	0.80	1.95	0.006	0.015
	KEGG_CELL_CYCLE	-0.62	-2.16	<0.0001	0.002
	KEGG_RNA_DEGRADATION	-0.61	-2.05	<0.0001	0.011
	KEGG_PROTEASOME	-0.75	-1.92	0.002	0.035
	KEGG_NUCLEOTIDE_EXCISION_REPAIR	-0.61	-1.92	0.002	0.029
	KEGG_BASAL_TRANSCRIPTION_FACTORS	-0.6	-1.88	0.002	0.039
	KEGG_DNA_REPLICATION	-0.74	-1.87	0.002	0.037

GSEA, Gene Set Enrichment Analysis; NES, normalized enrichment score; NOM, nominal; FDR, false discovery rate.

### External Verification of the GEO and THPA Databases and Immunohistochemistry

**Figure 9:**
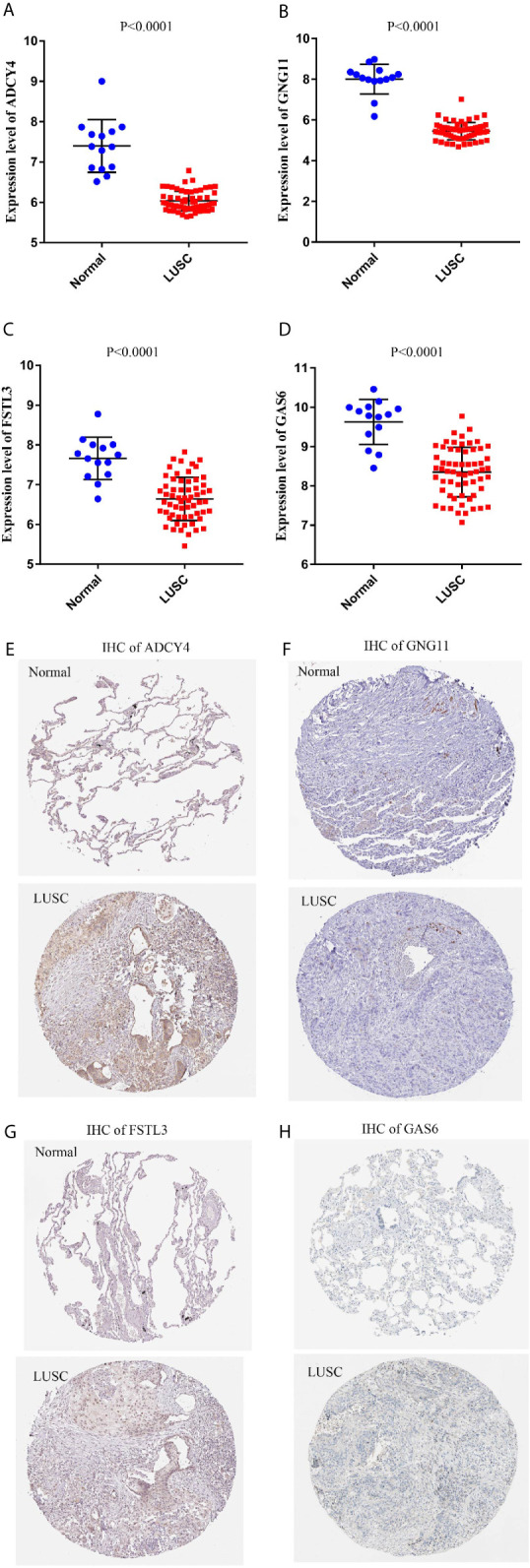
External validation of survival-related hub genes based on Gene Expression Omnibus (GEO) and the human protein atlas (THPA) databases. The mRNA expression patterns of **(A)** ADCY4, **(B)** GNG11, **(C)** FSTL3, and **(D)** GAS6 are compared between LUSC and normal lung tissues based on GSE30219. The protein expression patterns of **(E)** ADCY4, **(F)** GNG11, **(G)** FSTL3, and **(H)** GAS6 are compared between LUSC and normal lung tissues based on THPA database.

**Figure 10:**
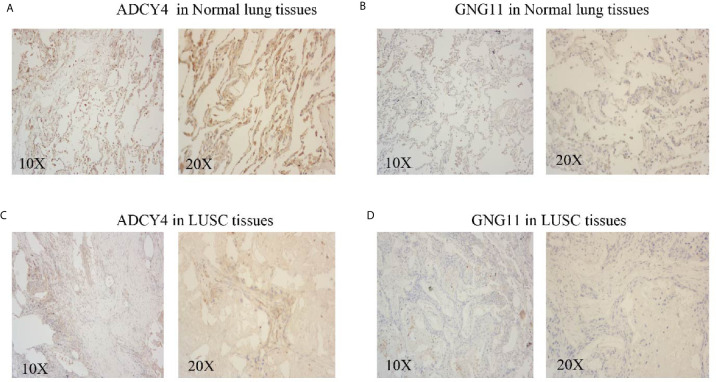
External validation of immunohistochemistry (IHC) outcomes from the real world. The **(A)** ADCY4 and **(B)** GNG11 expression levels in normal lung tissues. The **(C)** ADCY4 and **(D)** GNG11 expression levels in LUSC tissues.

To improve the reliability of our findings, external datasets were used for validation in this analysis. Firstly, we compared the mRNA expression levels of survival-related genes between LUSC and normal tissues using GSE30219. Compared with normal tissues, the mRNA expression of ADCY4 (**Figure 9A**), GNG11 (**Figure 9B**), FSTL3 (**Figure 9C**), GAS6 (**Figure 9D**) and CHRDL1 (**Figure S1A**) was lower in LUSC tissues. Secondly, the protein expression levels of OS-related genes were compared in LUSC and normal lung tissues using the THPA database. Although GNG11 was not detected in LUSC and normal lung tissues (**Figure 9F**), the protein expression patterns of ADCY4 (**Figure 9E**), FSTL3 (**Figure 9G**), GAS6 (**Figure 9H**) and CHRDL1 (**Figure S1B**) were consistent with their mRNA expression levels. **Table 2** illustrates the detailed information on IHC for the 5 OS-related genes between LUSC and normal lung tissues. Furthermore, IHC results from the real world demonstrated that ADCY4 was apparently downregulated in LUSC tissues, and GNG11 was not detected in LUSC and normal lung tissues (**Figure 10**), suggesting that ADCY4 probably plays an important role in LUSC.

**Table 2:** The detailed information of IHC results from THPA database.

**Gene**	**Normal lung tissues**								**LUSC tissues**			
	Macrophages				Pneumocytes				Tumor cells			
	Staining	Intensity	Quantity	Location	Staining	Intensity	Quantity	Location	Staining	Intensity	Quantity	Location
**ADCY4**	Medium	Moderate	75%-25%	Cytoplasmic/membranous	Medium	Moderate	75%-25%	Cytoplasmic/membranous	Low	Moderate	<25%	Cytoplasmic/membranous
**GNG11**	Not detected	Negative	None	None	Not detected	Negative	None	None	Not detected	Negative	None	None
**FSTL3**	Low	Moderate	<25%	Nuclear	Low	Weak	75%-25%	Nuclear	Low	Weak	75%-25%	Nuclear
**GAS6**	Low	Weak	>75%	Cytoplasmic/membranous	Not detected	Negative	None	None	Not detected	Negative	None	None
**CHRDL1**	Medium	Moderate	75%-25%	Cytoplasmic/membranous	Low	Weak	75%-25%	Cytoplasmic/membranous	Not detected	Negative	None	None

IHC, immunohistochemistry; THPA, The Human Protein Atlas; LUSC, lung squamous cell carcinoma.

## Discussion

As a prevalent malignant tumor with high mortality, lung cancer confers enormous socio-economic pressure on patients and families. Progress in the early diagnosis, treatment and predicted prognosis of LUSC is still limited. Therefore, it is urgent to find cancer-specific indicators for monitoring the progression and predicting the prognosis of LUSC patients. In this study, a total of 576 differentially co-expressed genes were found *via* integrated bioinformatics methods based on the GSE19188 and TCGA-LUSC datasets. Functional annotation analyses of these differentially co-expressed genes suggested that regulation of vasculature development, collagen−containing extracellular matrix, actin binding and PPAR signaling pathway were primarily enriched. Differentially co-expressed genes with the top ten highest MCC scores were designated as hub genes associated with LUSC. Subsequently, we observed that 5 hub genes (GNG11, ADCY4, FSTL3, GAS6, and CHRDL1) were highly correlated with the prognosis of LUSC patients. GSEA analysis illustrated that spliceosome, cell adhesion molecules, ECM receptor interaction and cytokine-cytokine receptor interactions were correlated with the expression of GNG11, ADCY4, FSTL3 and GAS6, respectively. Finally, based on the GSE30219 and THPA databases and IHC outcomes from the real world, we observed that although GNG11 was not detected, ADCY4 was significantly downregulated in LUSC tissues at the mRNA and protein levels.

ADCY4, adenylate cyclase 4, promoted the formation of the signaling molecule cAMP to respond to G-protein signaling (22). ADCY4 was found to be correlated with calcium signaling pathways, and intracellular Ca2+ activation might influence the carcinogenesis and adverse invasion of LUAD cells (23, 24). Several studies have reported that ADCY4 showed lower expression in various cancer tissues compared to normal tissues (25). In fact, few studies have reported the role of ADCY4 in cancer. ADCY4 is the core gene that is apparently downregulated in LUSC tissues (26). Similarly, Yu et al. revealed that ADCY was downregulated in LUAD tissues, and they demonstrated that ADCY4 was highly associated with overall survival among LUAD patients using the Kaplan-Meier plotter (27). In addition, Fan, et al. illustrated that ADCY4 was obviously downregulated in primary breast cancer (P<1.00e-12) compared to normal tissues, and this downregulation was closely correlated with ADCY4 promoter hypermethylation (28). Furthermore, IHC results from the real world validated the low expression of ADCY4 in LUSC compared to normal lung tissues. Given these outcomes, we believe that ADCY4 might be closely associated with the carcinogenesis and progression of LUSC, and ADCY4 may be a candidate therapeutic target and indicator to monitor progression and predict prognosis among LUSC patients.

Undeniably, there are some limitations of our study. (1) Although we conducted integrated bioinformatics analysis and IHC validation to select potential prognostic indicators in LUSC, this approach may not be extremely precise for patients with different LUSC stages and grades. (2) Though the GSE19188 and TCGA-LUSC datasets provided many samples of LUSC and non-malignant tissues for analysis, only the two datasets were included and analyzed. Additional related investigations are needed to further elucidate the role of ADCY4 in LUSC.

## Conclusion

In general, our analysis was conducted to find hub genes that may be correlated with the tumorigenesis and development of LUSC through differential gene expression analysis and WGCNA. Ten hub genes were selected according to the ranking of MCC scores, and five hub genes were apparently correlated with the prognosis of LUSC patients. Based on the GSE30219 and THPA databases and IHC results from the real world, we found that although GNG11 was not detected, ADCY4 was significantly downregulated in LUSC tissues. Thus, ADCY4 is a potential therapeutic and prognostic indicator in LUSC patients. However, more studies are needed to further verify and explore the biological relationships among these survival-related hub genes in LUSC.

## Data Availability Statement

The original contributions presented in the study are included in the article/**Supplementary Material**. Further inquiries can be directed to the corresponding author.

## Ethics Statement

The studies involving human participants were reviewed and approved by Huzhou Central Hospital. Written informed consent for participation was not required for this study in accordance with the national legislation and the institutional requirements.

## Author Contributions

ZL had full access to all of the data in the manuscript and takes responsibility for the integrity of the data and the accuracy of the data analysis. Concept and design: all authors. Acquisition, analysis, and interpretation of data: all authors. Drafting of the manuscript: all authors. Critical revision of the manuscript for important intellectual content: all authors. Statistical analysis: all authors. Supervision: LR and ZM. All authors contributed to the article and approved the submitted version.

## Funding

This study is supported by the Public Welfare Technology Application Research Program of Huzhou (No.2019GY35,2019GY01) and Young Talents Project of Huzhou Central Hospital (NO.2020YC09), without the involvement of commercial entities. The funder had no role in the design or performance of the study, the collection, management, analysis, and interpretation of the data, the preparation, review, and approval of the manuscript, or the decision to submit the manuscript for publication.

## Conflict of Interest

The authors declare that the research was conducted in the absence of any commercial or financial relationships that could be construed as a potential conflict of interest.

## Supplementary Material

Supplementary Figure 1 External validation of CHRDL1 based on Gene Expression Omnibus (GEO) and the human protein atlas (THPA) databases. The mRNA expression pattern of **(A)** CHRDL1 is compared between LUSC and normal lung tissues using GSE30219. The mRNA expression pattern of **(B)** CHRDL1 is compared between LUSC and normal lung tissues based on THPA database.

Supplementary Table 2 The differentially expressed genes (DEGs) in GSE19188.

Supplementary Table 3 The differentially expressed genes (DEGs) in TCGA-LUSC dataset.

Supplementary Table 4 The co-expression genes in the turquoise module of GSE19188.

Supplementary Table 5 The co-expression genes in the turquoise module of TCGA-LUSC dataset.

Supplementary Table 6 The intersection of genes among the two lists of differentially expressed genes (DEGs) and the two lists of co-expression genes.

## Abbreviations

NSCLC, non-small cell lung cancer; LUAD, lung adenocarcinoma; LUSC, lung squamous cell carcinoma; WGCNA, Weighed Gene Co-expression Network Analysis; DEGs, differentially expressed genes; GEO, Gene Expression Omnibus; TCGA, The Cancer Genome Atlas; THPA, The Human Protein Atlas; IHC, immunohistochemistry; FDR, false discovery Rate; TOM, topological overlap matrix; FPKM, fragments per kilobase per million; TPM, transcript per million; MCC, Maximal Clique Centrality; GO, gene ontology; KEGG, kyoto encyclopedia of genes and genomes pathway; PPI, protein-protein interaction network; STRING, Search Tool for the Retrieval of Interacting Genes; MCODE, Molecular Complex Detection; BP, biological processes; CC, cellular component; MF, molecular function; OS, overall survival; HR, hazard ratio; GSEA, Gene Set Enrichment Analysis; NES, Normalized enrichment score; NOM, nominal.
