# Supplementary figures and images for: Low Expression of ADCY4 Predicts Worse Survival of Lung Squamous Cell Carcinoma Based on Integrated Analysis and Immunohistochemical Verification
Source: Front Oncol. 2021 Jun 10;11:637733. doi: 10.3389/fonc.2021.637733 (PMC8225293; doi:10.3389/fonc.2021.637733)

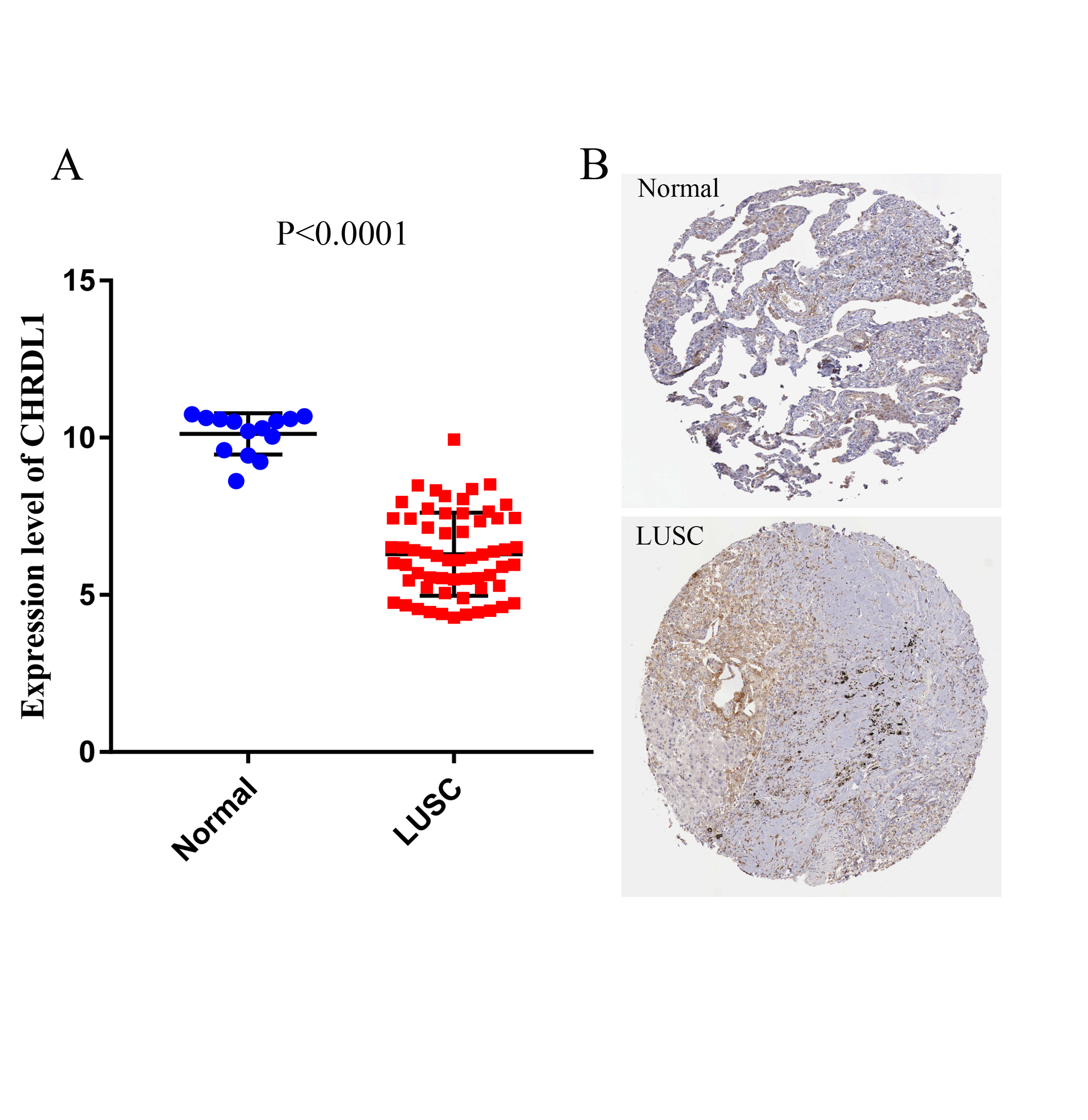

Supplement: Supplementary Figure 1 — External validation of CHRDL1 based on Gene Expression Omnibus (GEO) and the human protein atlas (THPA) databases. The mRNA expression pattern of (A) CHRDL1 is compared between LUSC and normal lung tissues using GSE30219. The mRNA expression pattern of (B) CHRDL1 is compared between LUSC and normal lung tissues based on THPA database. [file Image_1.tif]
