# Supplementary material for: Low Expression of ADCY4 Predicts Worse Survival of Lung Squamous Cell Carcinoma Based on Integrated Analysis and Immunohistochemical Verification
Source: Front Oncol. 2021 Jun 10;11:637733. doi: 10.3389/fonc.2021.637733 (PMC8225293; doi:10.3389/fonc.2021.637733)
Supplement: Supplementary file 2 [file Table_1.docx]

Table S1 Main demographic and clinicopathological characteristics of cases in TCGA-LUSC.

| Baseline characteristics | Value^*^ |
| --- | --- |
| **Age (years)** | 62.5 (39-74) |
| **Follow-up time (years)** | 1.81 (0-14.48) |
| **Gender** |  |
| Male | 373 (74.01) |
| Female | 131 (25.99) |
| **Radiation therapy** |  |
| Yes | 47 (9.33) |
| No | 350 (69.44) |
| Unknown | 107 (21.23) |
| **Neoadjuvant treatment** |  |
| Yes | 7 (1.39) |
| No | 495 (98.21) |
| Unknown | 2 (0.40) |
| KRAS mutation |  |
| Yes | 1 (0.20) |
| No | 14 (2.78) |
| Unknown | 489 (97.02) |
| **EGFR mutation** |  |
| Yes | 2 (0.40) |
| Unknown | 502 (96.60) |
| **Stage** |  |
| I | 245 (48.61) |
| II | 163 (32.34) |
| III | 85 (16.87) |
| IV | 7 (1.39) |
| Unknown | 4 (0.79) |
| **T stage** |  |
| T1 | 114 (22.62) |
| T2 | 295 (58.53) |
| T3 | 71 (14.09) |
| T4 | 24 (4.76) |
| **N stage** |  |
| N0 | 320 (63.49) |
| N1 | 133 (26.39) |
| N2 | 40 (7.94) |
| N3 | 5 (0.99) |
| Unknown | 6 (1.19) |
| **Metastasis** |  |
| Yes | 7 (1.39) |
| No | 414 (82.14) |
| Unknown | 83(16.47) |

**Abbreviations:** LUSC: lung squamous cell carcinoma; TCGA: The Cancer Genome Atlas.* values are expressed as median (range) or n (%).
