# Supplementary material for: Low Expression of ADCY4 Predicts Worse Survival of Lung Squamous Cell Carcinoma Based on Integrated Analysis and Immunohistochemical Verification
Source: Front Oncol. 2021 Jun 10;11:637733. doi: 10.3389/fonc.2021.637733 (PMC8225293; doi:10.3389/fonc.2021.637733)
Supplement: Supplementary file 8 [file Table_7.docx]

Table S7 Relative pathways associated with the expression of FSTL3 and GAS6 using GSEA.

| **Gene** | **Name** | **ES** | **NES** | **NOM p-value** | **FDR q-value** |
| --- | --- | --- | --- | --- | --- |
| FSTL3 | KEGG_SPLICEOSOME | -0.64 | -2.00 | <0.0001 | 0.020 |
|  | KEGG_CELL_CYCLE | -0.55 | -1.93 | 0.002 | 0.033 |
|  | KEGG_DNA_REPLICATION | -0.74 | -1.90 | <0.0001 | 0.034 |
|  | KEGG_RNA_DEGRADATION | -0.55 | -1.80 | 0.004 | 0.091 |
|  | KEGG_NUCLEOTIDE_EXCISION_REPAIR | -0.58 | -1.80 | 0.004 | 0.074 |
|  | KEGG_ECM_RECEPTOR_INTERACTION | 0.75 | 2.23 | <0.0001 | <0.0001 |
|  | KEGG_FOCAL_ADHESION | 0.61 | 2.16 | <0.0001 | <0.0001 |
|  | KEGG_HYPERTROPHIC_CARDIOMYOPATHY_HCM | 0.61 | 2.14 | <0.0001 | <0.0001 |
|  | KEGG_CYTOKINE_CYTOKINE_RECEPTOR_INTERACTION | 0.62 | 2.08 | <0.0001 | 0.002 |
|  | KEGG_COMPLEMENT_AND_COAGULATION_CASCADES | 0.74 | 2.05 | <0.0001 | 0.004 |
|  |  |  |  |  |  |
| GAS6 | KEGG_CYTOKINE_CYTOKINE_RECEPTOR_INTERACTION | 0.63 | 2.12 | <0.0001 | 0.003 |
|  | KEGG_LEUKOCYTE_TRANSENDOTHELIAL_MIGRATION | 0.59 | 2.09 | <0.0001 | 0.002 |
|  | KEGG_ECM_RECEPTOR_INTERACTION | 0.70 | 2.07 | <0.0001 | 0.002 |
|  | KEGG_SPLICEOSOME | -0.63 | -1.99 | 0.002 | 0.038 |
|  | KEGG_GLYCOSYLPHOSPHATIDYLINOSITOL_  GPI_ANCHOR_BIOSYNTHESIS | -0.66 | -1.92 | 0.002 | 0.055 |
|  | KEGG_ONE_CARBON_POOL_BY_FOLATE | -0.69 | -1.91 | <0.0001 | 0.041 |
|  | KEGG_HOMOLOGOUS_RECOMBINATION | -0.68 | -1.85 | 0.002 | 0.059 |
|  | KEGG_RNA_POLYMERASE | -0.62 | -1.84 | 0.006 | 0.056 |
|  | KEGG_BASAL_TRANSCRIPTION_FACTORS | -0.56 | -1.75 | 0.017 | 0.095 |
|  | KEGG_NUCLEOTIDE_EXCISION_REPAIR | -0.56 | -1.75 | 0.015 | 0.087 |

**Abbreviations:** GSEA: Gene Set Enrichment Analysis; NES: normalized enrichment score; NOM: nominal; FDR: false discovery rate.
